# PrP^Sc^ formation and clearance as determinants of prion tropism

**DOI:** 10.1371/journal.ppat.1006298

**Published:** 2017-03-29

**Authors:** Ronald A. Shikiya, Katie A. Langenfeld, Thomas E. Eckland, Jonathan Trinh, Sara A. M. Holec, Candace K. Mathiason, Anthony E. Kincaid, Jason C. Bartz

**Affiliations:** 1 Department of Medical Microbiology and Immunology, Creighton University, Omaha, Nebraska, United States of America; 2 Department of Microbiology, Immunology and Pathology, Colorado State University, Fort Collins, Colorado, United States of America; 3 Department of Pharmacy Science, Creighton University, Omaha, Nebraska, United States of America; Istituto Superiore di Sanità, ITALY

## Abstract

Prion strains are characterized by strain-specific differences in neuropathology but can also differ in incubation period, clinical disease, host-range and tissue tropism. The hyper (HY) and drowsy (DY) strains of hamster-adapted transmissible mink encephalopathy (TME) differ in tissue tropism and susceptibility to infection by extraneural routes of infection. Notably, DY TME is not detected in the secondary lymphoreticular system (LRS) tissues of infected hosts regardless of the route of inoculation. We found that similar to the lymphotropic strain HY TME, DY TME crosses mucosal epithelia, enters draining lymphatic vessels in underlying laminae propriae, and is transported to LRS tissues. Since DY TME causes disease once it enters the peripheral nervous system, the restriction in DY TME pathogenesis is due to its inability to establish infection in LRS tissues, not a failure of transport. To determine if LRS tissues can support DY TME formation, we performed protein misfolding cyclic amplification using DY PrP^Sc^ as the seed and spleen homogenate as the source of PrP^C^. We found that the spleen environment can support DY PrP^Sc^ formation, although at lower rates compared to lymphotropic strains, suggesting that the failure of DY TME to establish infection in the spleen is not due to the absence of a strain-specific conversion cofactor. Finally, we provide evidence that DY PrP^Sc^ is more susceptible to degradation when compared to PrP^Sc^ from other lymphotrophic strains. We hypothesize that the relative rates of PrP^Sc^ formation and clearance can influence prion tropism.

## Author summary

Strain specific distribution of prions throughout the infected host are observed in both naturally occurring and experimentally induced prion diseases. The distribution of prions in the host can influence prion shedding and transmission (e.g. iatrogenic prion transmission). The mechanism(s) responsible for strain tropism are unknown. Here we show that entry and transport of prions to lymphoid tissue are not influenced by the prion strain. However, we show that lymphotropic prion strains have a higher rate of PrP^Sc^ formation and a lower rate of prion degradation compared to a non-lymphotropic prion strain. We hypothesize that the relative rates of PrP^Sc^ formation and clearance is one of potentially several mechanisms that can determine prion strain tropism.

## Introduction

Prion diseases are infectious neurodegenerative diseases that affect animals including humans. Prion diseases of humans include Creutzfeldt-Jakob disease (CJD), Gerstmann-Straussler-Scheinker syndrome, fatal familial insomnia and kuru. Prion diseases of animals include scrapie of sheep and goats, bovine spongiform encephalopathy, TME, and chronic wasting disease (CWD) of cervids. All prion diseases are fatal and effective therapeutic treatments are not available. The infectious agent of these diseases is PrP^Sc^, a self-propagating isoform of the normal host prion protein, designated PrP^C^ [1–3]. In the absence of PrP^C^, the formation of new PrP^Sc^ is extinguished and preexisting PrP^Sc^ is cleared by an unknown mechanism [4–6]. Distinct strains of prions are characterized by differences in the distribution of spongiform degeneration in the central nervous system (CNS) [7,8].

The mechanism(s) by which PrP^Sc^ encodes strain diversity is unknown. Strain-specific conformations of PrP^Sc^ were first suggested by the observation of strain-specific Western blot profiles of PrP^Sc^ from murine adapted prion strains [9]. Further evidence was provided by the HY and DY strains of hamster-adapted TME. These two strains have distinct electrophoretic migration properties and conformational stability of PrP^Sc^ and, importantly, HY and DY PrP^Sc^ has strain-specific α-helical and β-sheet content [10–12]. Prion strains can have distinct PrP^Sc^ fibril structure and aggregate size suggesting strain-specific tertiary and quaternary structures [13,14]. It is unclear, however, if strain specific conformations of PrP^Sc^ are maintained by PrP^Sc^ alone or require a strain-specific cofactor. For example it is known that lipid molecules can influence the strain properties of *in vitro* amplified prion strains [15–17]. It has been hypothesized that the differences in the distribution of strain-specific prion cofactors in the host can influence which cells will support prion formation (i.e. tropism).

Prion strains are characterized by differences in tropism. The distribution of PrP^Sc^ in spleen and lymph nodes differs between sheep naturally infected with either the classical or the atypical strains of scrapie [18–20]. Similarly, in humans, a more widespread distribution of PrP^Sc^ in LRS tissues in variant CJD is observed as compared to classical CJD [21–23]. In natural prion disease, however, there are factors not controlled for that could explain the differences in PrP^Sc^ distribution unrelated to tropism (e.g. route of infection) [24–28]. In experimental prion disease where external variables are held constant, a more compelling example of strain-specific tissue tropism is observed. Hamsters infected with HY TME have detectable infectivity and/or PrP^Sc^ in the CNS, LRS, skeletal muscle, nasal secretions and blood [29–34]. In contrast, prion infectivity and/or PrP^Sc^ is restricted to the CNS of DY TME-infected hamsters [35–37]. Within a tissue or cell type, strain-specific differences in PrP^Sc^ distribution can occur. For example, hamsters or transgenic mice expressing hamster PrP^C^ infected with either the Sc237 or 139H strains of hamster-adapted scrapie are characterized by regional differences in the localization of PrP^Sc^ in the CNS [38,39]; and immunohistochemical detection of PrP^Sc^ can illuminate strain-specific differences in the cellular localization of PrP^Sc^ [40–43]. The mechanism(s) underlying prion tropism is unclear. In this study, we investigated the entry, transport, contributions of conversion cofactors and the rates of prion formation and clearance to prion tropism.

## Results

### In contrast to HY TME, extraneural DY TME agent inoculation fails to cause disease

Inoculation of hamsters with HY TME resulted in animals developing clinical signs of HY TME infection regardless of the route of infection (Table 1). Consistent with previous reports [35,37], inoculation of hamsters with DY TME in the central or peripheral nervous system (PNS) caused disease, while extraneural inoculation failed to cause disease at extended time points post infection (p.i.) (Table 1).

**Table 1:** Incubation period of disease in hamsters inoculated with either the HY or DY TME agent.

	Incubation period	
Route	HY TME	DY TME
Intracerebral	60±4 (5/5)	169±3 (10/10)^a^
Intranerve	68±3 (5/5)	236±3 (5/5)
Per os	158, 172 (2/5)	>600 (0/6)
Intraperitoneal	90±7 (5/5)	>600 (0/6)
Extranasal	194±43 (5/5)	> 650 (0/6)
Intramuscular	115±17 (5/5)	>550 (0/5)
Intravenous	n.d.	>500 (0/4)

^a^ days ± standard error of the mean to onset of clinical signs (number affected / number inoculated).

**Fig 1:**
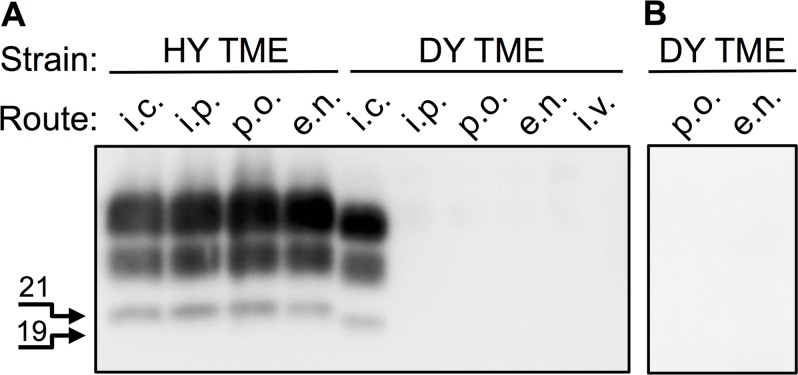
Extraneural inoculation of DY TME fails to establish infection in hamsters. Western blot analysis of proteinase K digested brain material from hamsters infected with either the HY or DY TME agents by various routes of inoculation. A) Inoculation of hamsters with the HY TME agent results in development of disease and detection of PrP^Sc^. B) Hamsters inoculated with DY TME a single (A) or multiple (B) times only develop disease following direct inoculation of the CNS. The 19 and 21 kDa unglycoslyated PrP^Sc^ polypeptides are indicated on the left of the panel. i.c., intracerebral; i.p., intraperitoneal; p.o., per os; e.n., extranasal; i.v., intravenous.

It is possible that the failure of DY TME to cause disease by extraneural routes of inoculation was due to low effective titer. To explore this possibility, hamsters were extranasally (e.n.) inoculated with DY TME to determine if a more efficient route of inoculation could cause disease. None of the DY TME (n = 6) or mock-inoculated (n = 3) hamsters developed clinical signs of DY TME at 650 days p.i. when the experiment was terminated (Table 1). Western blot analysis of 250 μg equivalents of proteinase K digested brain material from these animals failed to detect PrP^Sc^ (Fig 1, panel A). The olfactory bulb, trigeminal ganglia, cervical lymph nodes and Peyer’s patches were collected from two DY TME e.n. infected and one mock e.n. infected hamster. Each of the tissues was homogenized and the entire homogenate was intracerebrally (i.c.) inoculated into 4 hamsters to determine if the DY TME agent was present. The recipient hamsters failed to develop clinical signs of DY TME at 400 days p.i. when the experiment was terminated (Table 2). Western blot analysis of 125 μg equivalents of proteinase K digested 5% w/v brain material from these animals failed to detect PrP^Sc^ (S1 Fig). As a positive control, hamsters were i.c. inoculated with the DY TME agent. All (n = 10) of the animals developed clinical signs of progressive lethargy consistent with DY TME agent infection at 169±3 days p.i.. Western blot analysis of 125 μg equivalents of proteinase K digested brain material from these animals detected PrP^Sc^ with DY TME migration properties confirming the clinical diagnosis (Fig 1, panel A).

**Table 2:** TME agent infectivity in tissues from hamsters extranasally inoculated with the DY TME agent.

Inoculation^a^	Time post inoculation (days)	Incubation period (days)			
		Olfactory bulb	Trigeminal ganglion	Cervical lymph node	Peyer’s Patch
Mock	n.a.	>400 (0/4)^b^	>400 (0/4)	>400 (0/4)	>400 (0/4)
DY TME–Ha 1	650	>400 (0/4)	>400 (0/4)	>400 (0/4)	>400 (0/4)
DY TME–Ha 2	650	>400 (0/4)	>400 (0/4)	>400 (0/4)	>400 (0/4)

^a^ Hamsters were extranasally inoculated with 10ul of a 1% w/v brain homogenate containing 10^7.4^ i.c. LD_50_/g of the DY TME agent or a normal brain homogenate (mock). The olfactory bulb, trigeminal ganglion, cervical lymph node and Peyer’s patch were collected from a mock-inoculated and two DY TME agent-inoculated hamsters (Ha 1 and Ha 2) at the indicated times post-inoculation for animal bioassay.

^b^ number affected / number inoculated.

n.a.–not applicable.

Repeated inoculations are known to increase the efficiency of prion infection [44,45]. To investigate if repeated inoculations of DY TME could cause disease groups (n = 5) of hamsters were inoculated with DY TME by either the e.n. or per os route once per week for 10 weeks total. These hamsters failed to develop clinical signs of DY TME at 650 days p.i. following the first infection when the experiment was terminated (Table 2). Western blot analysis of 125 μg equivalents of proteinase K digested 5% w/v brain material from these animals failed to detect PrP^Sc^ (Fig 1, panel B). Overall, DY TME fails to cause disease by extraneural routes of inoculation.

### Rapid transepithelial transport and detection of DY TME prionemia by extranasal infection

**Fig 2:**
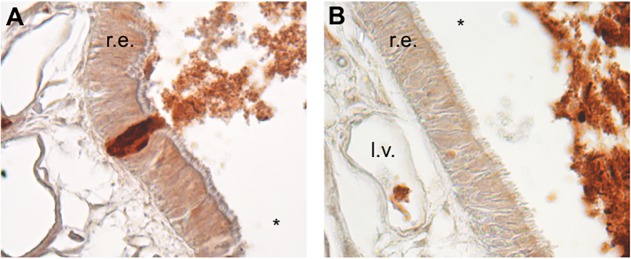
Rapid transepithelial transport following extranasal infection with DY TME. Hamsters (n = 3) were extranasally inoculated with DY TME agent and at 10 minutes post infection inoculum was in the airway (*), crossing the (A) respiratory epithelia (r.e.) and in (B) the lumen of lymphatic vessels (l.v.) in the lamina propriae.

Prion strains that cause disease via e.n. infection rapidly cross the respiratory and olfactory epithelia that line the surfaces of the nasal cavity (NC) [46–48]. We performed IHC on the NC of DY TME and mock-infected hamsters at 10 minutes post infection to determine if the inability of the DY TME agent to cause disease following e.n. infection was due to an inability to cross the nasal epithelia. We found DY TME-infected brain homogenate and PrP^Sc^ in the luminal airspace of the NC of all 3 animals (Fig 2, panels A and B) and there were examples of DY TME-infected brain homogenate traversing the epithelia of the NC in all 3 animals (Fig 2, panel A). The inoculum was located between intact cells consistent with intercellular transport (Fig 2, panel A). In all infected animals, DY TME-infected brain homogenate was also detected in the lumen of lymphatic vessels located in the lamina propria of all 3 animals (Fig 2, panel B). A similar pattern of brain homogenate distribution was observed in both (n = 2) of the mock-infected hamsters, which is consistent with previous results [47].

### Transport of DY PrP^Sc^ to lymphoreticular system tissues is not impaired

**Fig 3:**
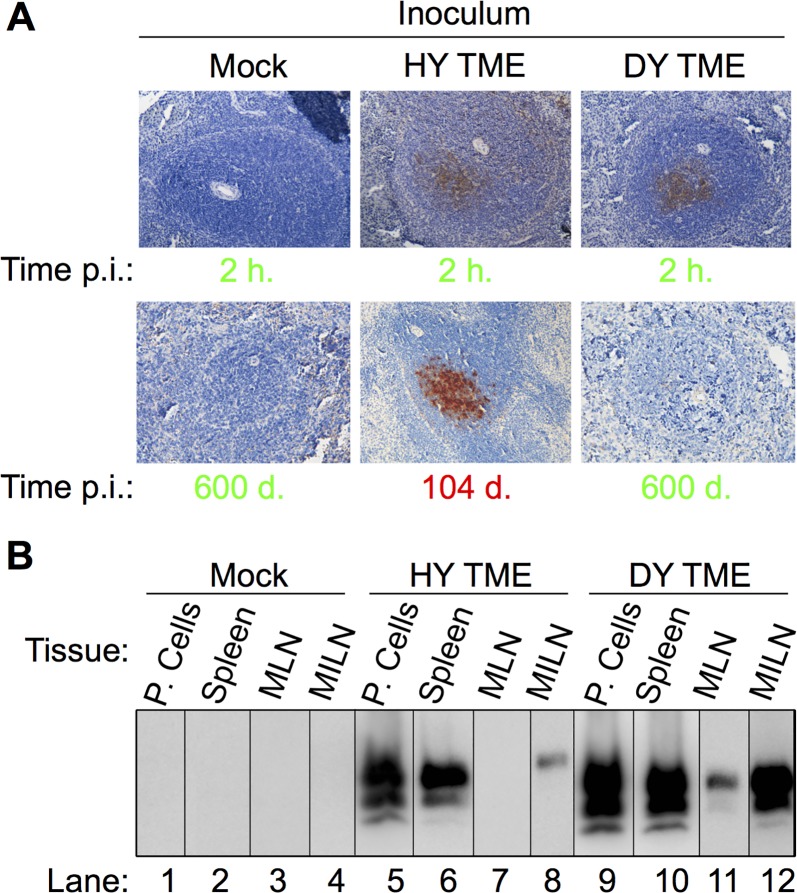
Rapid transport of HY and DY PrP^Sc^ to lymphoid tissues following intraperitoneal inoculation. Hamsters (n = 3 per inoculum) were intraperitoneally inoculated with brain homogenate from uninfected (Mock), HY TME or DY TME-infected hamsters and PrP^Sc^ was detected with either A) immunohistochemistry or B) Western blot. A) Spleen from negative control mock inoculated animals did not contain detectable PrP^Sc^ at 2 hours p.i. or at 600 d. p.i.. Spleen from positive control HY TME infected hamsters contained PrP^Sc^ immunoreactivity in the germinal center of lymphoid follicles at both 2 hours p.i. and during the clinical phase of disease. PrP^Sc^ immunoreactivity was detected in spleen from DY TME infected hamsters at 2 hours p.i. but not at 600 days p.i.. Incubation periods in green text are clinically normal and in red text are clinically affected. B) Western blot of peritoneal cells (P. Cells), spleen, mesenteric lymph node (MLN) and medial iliac lymph node (MILN) from hamsters intraperitonally inoculated with uninfected (Mock), HY TME or DY TME-infected brain homogenate at 2 hours p.i.

To investigate if DY PrP^Sc^ transport to secondary LRS tissues is inhibited, hamsters (n = 3 per group) were i.p. inoculated with either uninfected, HY TME or DY TME-infected brain homogenate. PrP^Sc^ IHC performed on spleen from animals at 2 hours p.i. failed to detect PrP^Sc^ in the mock inoculated group, while PrP^Sc^ was detected in animals inoculated with either HY or DY TME (Fig 3, panel A). PrP^Sc^ IHC of spleen from HY TME inoculated animals in the clinical phase of disease (104 d. p.i.) contained PrP^Sc^ immunoreactivity (Fig 3, panel A). Hamsters inoculated with DY TME and age matched mock infected controls did not contain detectable PrP^Sc^ immunoreactivity at 600 days p.i. (Fig 3, panel A). Peritoneal lavage cells (P. Cells), spleen, mesenteric lymph node (MLN) and medial iliac lymph node (MILN) from hamsters (n = 3 per group) i.p. inoculated with uninfected (Mock), HY TME, or DY TME-infected brain homogenate (Fig 3, panel B) at 2 hours p.i. was analyzed for the presence of PrP^Sc^ by Western blot. In the mock-inoculated negative control group, all tissues examined failed to detect PrP^Sc^ (Fig 3, panel A, lanes 1–4). In the positive control HY TME infected group, PrP^Sc^ was detected in P. cells, spleen and MILN, but was not detected in MLN (Fig 3, panel B, lanes 5–8). In the DY TME infected animals, PrP^Sc^ was detected in all lymphatic tissues examined (Fig 3, panel B, lanes 9–12). Overall, DY PrP^Sc^ is transported to secondary LRS tissues following i.p. inoculation.

### Spleen environment supports formation of HY, 139H and DY PrP^Sc^

**Fig 4:**
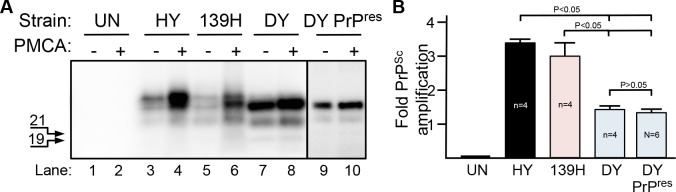
Lymphoid tissue supports DY PrP^Sc^ formation *in vitro*. Western blot (A) and quantification (B) of PMCA reactions containing hamster spleen homogenate as the template for conversion were seeded with brain homogenate (lanes 1–8) or detergent enriched, PK digested PrP-res (lanes 9–10) from uninfected (UN), 139H scrapie (139H), HY TME (HY) or DY TME (DY) infected hamsters. Reactions before (-) or after (+) PMCA are shown. The 19 and 21 kDa unglycoslyated PrP^Sc^ polypeptides are indicated on the left of the panel.

PMCA supports 139H, HY and DY TME PrP^Sc^ formation and agent replication when BH is the template for conversion [41]. To investigate if PMCA supports prion formation using spleen homogenate as the template for conversion, PMCA reactions (n = 4 per group) were spiked with BH from either HY TME-infected or 139H-infected animals as a positive control, mock-infected BH as a negative control or DY TME-infected BH or enriched PK digested DY PrP-res (Fig 4; S2 Fig). All samples were digested with PK prior to Western blot analysis. A significant (p<0.05) fold increase in PrP^Sc^ abundance (Fig 4, panel A, lanes 4 and 6) was observed in the HY TME (3.16±0.14) and 139H (3.02±0.41) seeded groups compared to the starting material following one round of PMCA (Fig 4, panel A, lanes 3 and 5) indicating that LRS tissue can support PrP^Sc^ formation similar to what has been observed *in vivo* [27,49]. A significant (p<0.05) 1.33±0.09 and 1.29±0.11 fold increase in PrP^Sc^ abundance was observed in PMCA reactions seeded with DY TME brain homogenate (Fig 4, panel A, lane 8) or detergent enriched PK digested DY PrP-res (Fig 4, panel A, lane 10) respectively, compared to the starting reactions (Fig 4, panel A, lanes 7 and 9). PrP^Sc^ was not detected in the negative control PMCA reactions (Fig 4, panel A, lane 2). The migration of the spleen PMCA generated and DY PrP^Sc^ was 1–2 kDa faster compared to HY and 139H similar to what is observed in brain-derived PrP^Sc^ (Fig 4, panel A). The fold PrP^Sc^ abundance of HY and 139H seeded reactions was similar (p>0.05) and were significantly different (p<0.05) compared to DY TME brain homogenate or PrP-res seeded reactions (Fig 4, panel B). The fold PrP^Sc^ abundance of DY TME brain homogenate and DY PrP-res seeded reactions did not significantly (p>0.05) differ (Fig 4, panel B). Overall, spleen contained all of the necessary components for HY, 139H and DY PrP^Sc^ formation and the levels of PrP^Sc^ formation for the lymphotropic strains HY TME and 139H were greater compared to the non-lymphotropic strain, DY TME. This experiment was repeated at least seven times with similar results.

### Strain specific sensitivity of PrP^Sc^ to proteolytic degradation

**Fig 5:**
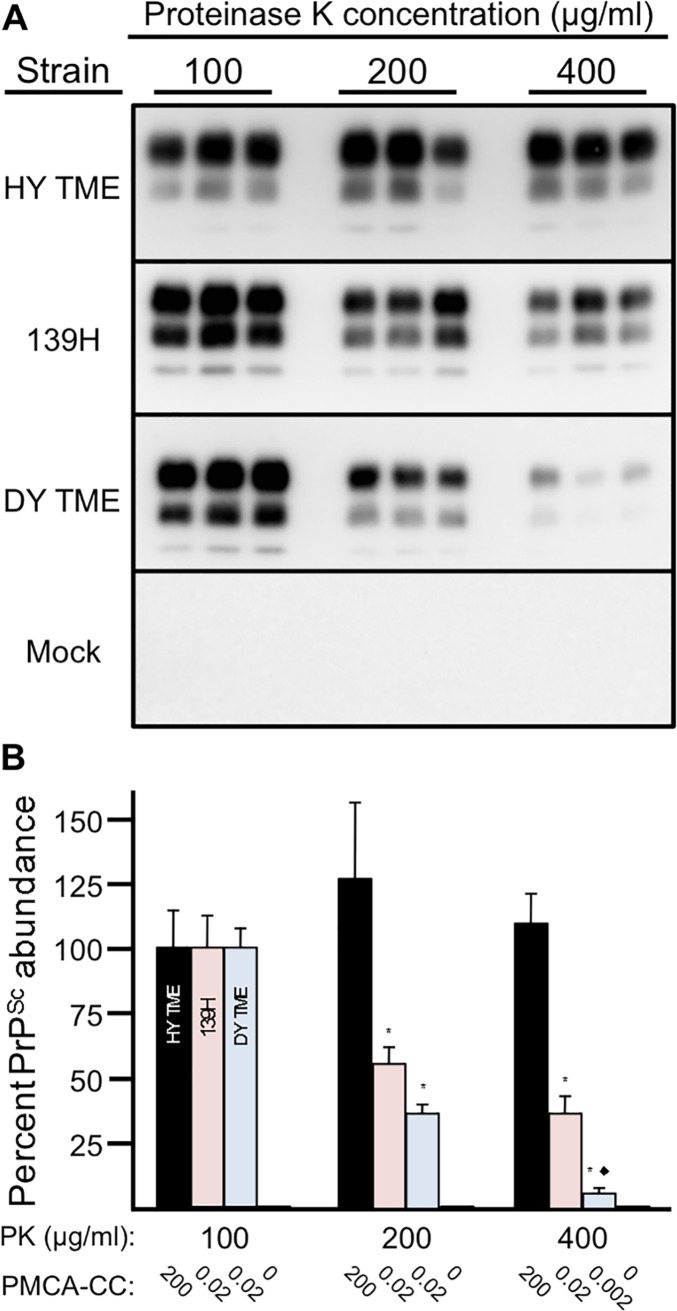
Strain-specific sensitivity of PrP^Sc^ to degradation. Western blot (A) and quantification and PMCA converting activity (B) of proteinase K digested brain homogenate from mock-infected (mock) or hamsters infected with either the HY TME, 139H or DY TME agents. *Significantly different (p<0.05) from 100 μg/ml treated material. ◆Significantly (p<0.05) different compared to 200 μg/ml 139H, 400 μg/ml 139H and 200 μg/ml DY TME.

To investigate the relative strain-specific susceptibility of PrP^Sc^ to proteolytic degradation, 2.5% w/v BH collected from animals at the terminal stage of HY TME, DY TME, or 139H infection (n = 3 per group) were subject to incubation with increasing concentrations of PK. BH from uninfected hamsters (n = 3) was used as a negative control. The amount of PrP in the 100 μg/ml PK group was normalized to 100 percent (Fig 5, panel B). The percentage of HY, 139H and DY PrP^Sc^ in the 200 μg/ml PK group was 127±30, 56±6 and 37±4 respectively (Fig 5, panel B) and the percentage of HY, 139H and DY PrP^Sc^ in the 400 μg/ml PK group was 110±12, 37±7 and 6±2 respectively (Fig 5, panel B). The abundance of HY PrP^Sc^ was similar at all PK concentrations tested (p>0.05). The abundance of 139H PrP^Sc^ with 200 and 400 μg/ml of PK was similar (p>0.05) but significantly (p<0.05) different than 139H PrP^Sc^ treatment with 100 μg/ml of PK. The abundance of DY PrP^Sc^ was decreased significantly (p<0.05) with each PK concentration tested. The PMCA conversion coefficient (PMCA-CC) of HY PrP^Sc^ and 139H PrP^Sc^ was similar at all PK concentration tested. The PMCA-CC for DY PrP^Sc^ was similar for the 100 and 200 μg/ml PK groups but was reduced 10 fold in the 400 μg/ml PK treatment group. PrP^Sc^ and PMCA seeding activity were not detected in the mock treated groups (Fig 5). Overall, DY PrP^Sc^ was more susceptible to proteolytic degradation with a corresponding decrease in PMCA-CC compared to lymphotropic strains.

## Discussion

Extraneural inoculation of DY TME infected BH failed to cause disease even via highly efficient extraneural routes of infection and following repeated inoculations. Previous studies have shown that a single inoculation of DY TME fails to cause disease by the per os, i.p., intravenous or intramuscular routes of infection (Fig 1; Table 1; [27,35,36]. These extraneural routes of inoculation are, in general, orders of magnitude less efficient at causing disease compared to i.c. inoculation [25,50–52]. The i.c. LD_50_ of DY TME is 100 fold lower compared to HY TME, therefore, DY TME may fail to cause disease due to a low effective titer [32]. In an attempt to increase the effective titer of DY TME, we used e.n. inoculation and repeated inoculations of prions [44, 45,50,53–55]. We found that e.n. inoculation of DY TME failed to cause disease within the lifespan of the host consistent with a previous report [35]. Importantly, bioassay of selected LRS, PNS and CNS tissues collected from these animals failed to detect DY TME agent, indicating that a subclinical infection had not been established consistent with previous findings following i.p. inoculation of DY TME (Fig 1; Table 2; [56]). Repeated inoculations of DY TME by either the per os or e.n. routes failed to cause clinical signs of DY TME infection within 650 days of the first inoculation. Taken together this transmission data suggests that the failure of DY TME to cause disease or establish infection was not due to effective titer, however, we can not exclude the possibility that increasing the sample size for each group would identify a rare positive transmission event. Overall, these studies suggest that other factor(s) control this well characterized example of an exclusively neurotropic prion strain.

**Fig 6:**
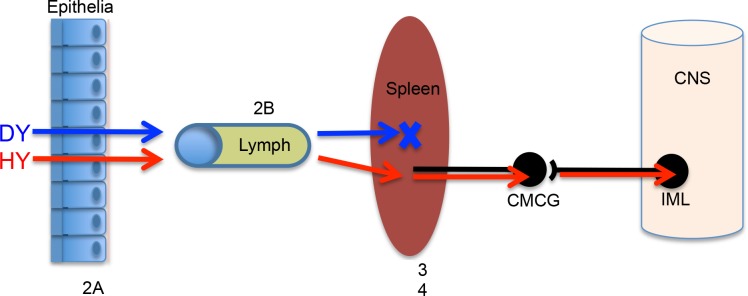
Model of prion strain transport. Following peripheral inoculation of HY TME (HY, Red arrows) or DY TME (DY, Blue arrows), PrP^Sc^ is transported across the epithelia, enters draining lymphatic circulation and is transported to secondary lyphoreticular system (LRS) tissues. At this point, HY TME establishes a productive infection, enters the peripheral nervous system and is retrogradely transported to the CNS where it causes clinical disease. In contrast, DY TME fails to establish infection in secondary LRS tissues, however, inoculation of peripheral nerves with DY TME results in transport to the CNS and establishment of infection indicating that the restriction in DY pathogenesis occurs in secondary LRS tissues. Since these tissues contain the necessary factors for DY TME formation *in vitro*, the relatively low rate of PrP^Sc^ formation and high rate of PrP^Sc^ degradation, compared to HY PrP^Sc^ may contribute to tissue tropism and the failure to establish disease. CMCG—celiac and mesenteric ganglion complex. IML—intermediolateral cell column of the mid thoracic spinal cord. X–Location of restriction of DY TME pathogenesis.

DY TME can be found in LRS tissues within hours of inoculation. Previous studies have failed to detect DY PrP^Sc^ or infectivity in spleen and lymph nodes at later (e.g. months) time points p.i. [36]. We hypothesized that DY TME inoculum fails to reach spleen and lymph nodes resulting in an inability to cause disease. However, we found that DY PrP^Sc^ crossed the nasal mucosal epithelium and was detected in the lumen of lymphatics at 1 hour p.i. (Fig 2). This data indicates that DY TME can cross epithelia and enter the circulation adding to the growing body of literature indicating that entry of PrP^Sc^ into or between cells occurs independent of the prion strain [47]. Additionally, DY PrP^Sc^ was detected in secondary LRS tissues following i.p. inoculation at 2 hours p.i. (Fig 3). This data indicates that cellular transport of prions from the point of inoculation to the spleen did not degrade DY PrP^Sc^. We interpret this to be detection of inoculum PrP^Sc^, however, we can not exclude the possibility that newly formed PrP^Sc^ is contributing to the results. The localization of HY and DY PrP^Sc^ in germinal centers of spleen was similar (Fig 3) suggesting similar targeting. We hypothesize that strain specific differences in PrP^Sc^, such as sialylation, does not affect the initial targeting, which is consistent with previous work [57,58], however, the contribution of sialylation to newly formed PrP^Sc^ in the spleen is not known [59]. We can not exclude the possibility that strain-specific requirements for cellular entry explain the failure to establish infection, however, prion uptake studies in cell culture are not consistent this hypothesis [60,61]. Detailed analysis of the early events in HY and DY TME pathogenesis failed to identify strain specific differences in transport to secondary LRS tissues. These observations are inconsistent with the hypothesis that strain-specific differences in prion transport result in a failure of DY TME to establish infection in spleen and lymph nodes. Furthermore, this data indicates that the restriction in DY pathogenesis must be occurring in secondary LRS tissues (Fig 6).

The spleen supports DY PrP^Sc^ formation. Cellular conversion cofactors can change strain properties of *in vitro* generated PrP^Sc^ suggesting that tropism could be influenced by the distribution of host strain-specific conversion cofactors [15–17]. This hypothesis predicts that the failure of DY TME to establish infection in the LRS is caused by a lack of DY PrP^Sc^ specific host cofactor(s) required for prion conversion. We used PMCA to directly test this hypothesis and found that DY PrP^Sc^ formation was supported in spleen homogenate (Fig 4). We found that both DY TME brain homogenate and detergent enriched PK-digested DY PrP-res supported PrP^Sc^ formation indicating that residual PrP^C^ or conversion cofactors contained in the DY brain homogenate seed did not contribute to the formation of DY PrP^Sc^ (Fig 4, S2 Fig). Additionally, the observation that the conversion activity of DY TME brain homogenate and DY PrP-res are similar suggests that sensitive forms of DY PrP^Sc^ do not significantly contribute to the observed results (Fig 4, S2 Fig). This indicates that all of the factors, including PrP^C^ that supports conversion by DY PrP^Sc^, required to amplify DY PrP^Sc^ are contained within the spleen [62]. These findings are consistent with *in vitro* cell-free conversion experiments demonstrating that RK13 and baculovirus derived PrP^C^ can support DY PrP^Sc^ formation [63,64]. These data suggest that all of the DY strain-specific information is contained within the structure of PrP^Sc^ [10,65]. We can not exclude the possibility that cell types in the spleen that amplify PrP^Sc^ (e.g. follicular dendritic cells) lack DY strain-specific conversion cofactor(s) and therefore do not support DY PrP^Sc^ formation *in vivo*. In this scenario the DY strain specific co-factor would have to be present in other cells in the spleen that provide the conversion cofactor *in trans* during PMCA.

Strain-specific rates of PrP^Sc^ accumulation (i.e. that balance between formation and clearance) occur *in vivo* [28,66]. This has been further investigated with PMCA that supports PrP^Sc^ formation but not clearance [67]. Using PMCA with brain homogenate as the template for conversion, prions strains with short incubation periods generally have more efficient PrP^Sc^ formation compared to PrP^Sc^ from long incubation period strains [41,68]. Using brain as the source of PrP^C^, HY TME amplifies more efficiently compared to 139H and DY TME which have the same, lower, amplification efficiency [41]. Interestingly, in this study, we found that using spleen homogenate as the template for PMCA conversion, HY TME and 139H have a similar rate of PrP^Sc^ formation that is greater than the amplification efficiency of DY TME (Fig 4, panel B). This data indicates that DY TME, while it is able to form new PrP^Sc^, does so less efficiently in the spleen compared to these two lymphotropic strains. This data also indicates that both the prion strain and host tissue can influence the efficiency of PrP^Sc^ formation. In brain, 139H and DY TME has the same PMCA conversion efficiency, can establish infection in the brain and have similar profiles of PrP^Sc^ deposition in neurons [41]. In spleen, 139H has a more efficient PMCA conversion efficiency compared to DY TME and 139H can establish infection in the spleen while DY TME does not (Fig 4). Assuming that all prion strains have similar rates of clearance, this data would suggest that the failure of DY TME to establish infection in the spleen is that the rate of formation does not exceed the rate of clearance [69]. We found that the susceptibility of brain derived DY PrP^Sc^ to degradation is greater than that of the lymphotropic strains 139H and HY TME (Fig 5) further suggesting that the balance between replication and clearance favors clearance of DY TME. A limitation of this observation is that brain derived PrP^Sc^ may have a different relative conversion efficiency and PK sensitivity compared to PrP^Sc^ produced in the spleen. Additionally, strain and cell differences in uptake and PrP^Sc^ disaggregation are not taken into account [60,61,70]. Clearly more work is needed to understand the relationship between the strain-specific features of PrP^Sc^ and host cell interactions.

Prion tropism is quite unlike viral tropism, which is largely influenced by the distribution of viral receptors. Our data indicates that two prion strains can similarly cross epithelia, enter the circulation, and are rapidly transported to secondary LRS tissues (Fig 6). This lack of specificity in prion transport implies that prions are widely distributed throughout the host following inoculation. Since PrP^C^ expression is widespread throughout the host, mechanism(s) must account for the limited distribution of prions. The strain-specific distribution of conversion cofactors could account for this observation. Additionally, the results presented here lead us to hypothesize that the balance between PrP^Sc^ formation and clearance can also influence the distribution of prions in the host.

## Materials and methods

### Ethics statement

All procedures involving animals were approved by the Creighton University Institutional Animal Care and Use Committee (protocol numbers 811 and 880) and comply with the *Guide for the Care and use of Laboratory Animals*.

### Prion strains

The HY and DY strains of hamster-adapted TME and the 139H strain of hamster-adapted scrapie were used in this study [41]. The titer for each biologically cloned TME strain was determined by end-point dilution of brain homogenate (BH) from terminally-ill animals. The titer of HY TME and DY TME agents were 10^9.3^ i.c. LD_50_ and 10^7.7^ i.c. LD_50_ per gram of brain, respectively [71].

### Animal inoculations

Male Syrian hamsters (Harlan-Sprague-Dawley, Indianapolis, IN) were used. Animals were inoculated a single time by the intracerebral (25μl of a 1% w/v BH), intraperitoneal (100μl of a 10% w/v BH), per os (100μl of a 10% w/v BH), extranasal (20μl of a 10% w/v BH), intranerve (2μl of a 1% w/v BH) or intravenous route (2μl of a 1% w/v BH) as described previously [37,72]. For the multiple inoculation studies, hamsters were inoculated once per week per os (100μl of a 1% w/v BH) or extranasally (20μl of a 1% w/v BH) for a total of 10 weeks. Hamsters were observed three times per week for the onset of clinical signs and the incubation period was calculated as the number of days between inoculation and the onset of clinical disease.

### Tissue collection

Prion-infected and age matched mock-infected hamsters were anesthetized with isoflurane and transcardially perfused with 50ml of 0.01 M Dulbecco’s phosphate buffered saline (DPBS) followed by 75ml of McLean’s paraformaldehyde-lysine-periodate (PLP) fixative for the experiments that utilized immunohistochemistry [73]. The skull was immediately removed and immersed in PLP for 5–7 hours and decalcified (Richard Allan, Kalamazoo, MI) for 14 days at room temperature prior to paraffin processing. For protein misfolding cyclic amplification experiments, uninfected hamsters were anesthetized with isoflurane and transcardially perfused with 75 ml of ice-cold phosphate buffer saline containing 5 mM EDTA, pH 7.4. Brain and spleen were immediately collected, frozen on dry ice and stored at -80°C. For Western blot analysis, animals were euthanized with CO_2_ followed by thoracotomy. Brain tissue was immediately collected, frozen on dry ice and stored at -80°C.

### Enrichment of PrP^Sc^

PrP^Sc^ was enriched by the method of Wenborn with the addition of PK [74]. Briefly, brain homogenate were digested with 10mg/ml of Pronase E followed by digestion with 50U/ml of Benzonase in 2% w/v sarkosyl (NLS). Sodium phosphotungstic acid (NaPTA) was added to 4% w/v and the samples were incubated at 37°C for 30 minutes. The samples were adjusted to 35%w/v iodixanol and 0.3% w/v NaPTA and centrifuged at 16,000xg for 90 minutes. The clarified supernatant was collected and filtered with a 0.45μm microcentrifuge filtration unit. The filtrate was mixed with an equal volume of 2% w/v NLS and 0.3% w/v NaPTA, incubated for 10 min and centrifuged at 16,000xg for 90 minutes. The pellet was resuspended in wash buffer (17.5% w/v iodixanol and 0.1% w/v NLS) and was digested with PK at 0.1mg/ml for 60 min at 37°C that was terminated using 10mM pefabloc. The final washing of the samples was performed by the addition of wash buffer followed by centrifugation at 16,000xg for 30 minutes. This process was repeated two times and the final pellet was resuspended in 0.1% w/v NLS. Western blot analysis was used to determine the abundance of PrP^Sc^ and the volume was adjusted to give the same abundance of PrP^Sc^ as in a 10% w/v brain homogenate. The enriched PrP-res was analyzed by Sypro Ruby staining to determine purity and PMCA to determine converting activity as previously described [75].

### Immunohistochemistry

Immunohistochemistry was performed as previously described [76]. Briefly, 7μm tissue sections were deparaffinized and incubated in 95% formic acid (Sigma-Aldrich, St. Louis, MO) for 20 minutes at room temperature. Endogenous peroxidase activity was blocked using 0.3% H_2_O_2_ in methanol for 20 minutes at room temperature. Non-specific staining was blocked with 10% normal horse serum (Vector Laboratories, Burlingame, CA) in tris-buffered saline (TBS) for 30 minutes at room temperature. The sections were incubated with anti-glial fibrillary acidic protein (GFAP; 1:16,000; Dako; Carpinteria, CA) at 4°C overnight. The sections were incubated in either a biotinylated horse anti-mouse or anti-rabbit immunoglobulin G conjugate and subsequently incubated in ABC solution (Elite kit; Vector Laboratories, Burlingame, CA). Sections were developed using 0.05% w/v 3,3’-diaminobenzidine (Sigma-Aldrich, St. Louis, MO) in TBS containing 0.0015% H_2_O_2_ and counterstained with hematoxylin (Richard Allen Scientific, Kalamazoo, MI). Light microscopy was performed using a Nikon i80 microscope (Nikon, Melville, NY) and images were captured and processed using Adobe Photoshop CS6 (San Jose, CA) using identical parameters.

### Protein misfolding cyclic amplification (PMCA)

Experiments using PMCA were performed as previously described [75]. Briefly, HY TME, DY TME or 139H-infected brains were homogenized to 10% w/v in DPBS (Mediatech, Herndon, VA) or enriched PrP^Sc^ was used as a PMCA seed. Uninfected brain and spleen tissues were homogenized to 10% w/v and 20% w/v in conversion buffer respectively, and used as a PMCA substrate. PMCA seeds were diluted in PMCA substrate at a 1:100 ratio e.g. 1 μl of seed was diluted into 99 μl of substrate. PMCA was performed with a Misonix 4000 sonicator (Farmingdale, NY). For PMCA with uninfected brain as substrate the sonicator output set to level 75 and an average power output of 160 watts during each sonication cycle. For PMCA with uninfected spleen as substrate, the sonicator output was set to level 87 with an average power output of 220 watts during each sonication cycle. One round of PMCA consist of 144 cycles; one cycle comprises of five-second sonication followed by a ten-minute incubation at 37°C. All PMCA sample groups had an n = 3 and all experiments were replicated a minimum of three times.

### Western blot analysis

Western blot detection of PrP^Sc^ from brain homogenate was performed as described previously [76]. Briefly, brain homogenate (5% w/v) in PMCA conversion buffer is digested with proteinase K (PK) at a final concentration of 100, 200, or 400 μg/ml (Roche Diagnostics Corporation, Indianapolis, IN) at 37°C for 1 or 24 hours. The samples were either enriched for PrP^Sc^ as described previously [77] or an equal amount of sample buffer containing 4% (v/v) 2-mercapto ethanol and 8% (w/v) SDS was added and the mixture was incubated at 100°C for 10 minutes. Prion protein was detected with the anti-PrP antibody 3F4 (final concentration of 0.1 μg/ml; Chemicon; Billerica, MA) and HRP-conjugated donkey anti-mouse secondary antibody (Jackson ImmunoResearch; West Grove, PA). The Western blot was developed with Pierce Supersignal West Femto Maximum Sensitivity Substrate according to manufacturer instructions (Pierce, Rockford, IL), imaged on a Kodak 4000R Imaging Station (Kodak, Rochester, NY) and analyzed using Kodak Molecular Imaging Software v.5.0.1.27 (New Haven, CT). Statistical analysis was performed using Prism 6.0 for Mac (GraphPad Software Inc., La Jolla, CA).

## Supporting information

S1 Fig Extranasal inoculation of DY TME does not establish prion infection. Western blot analysis of proteinase K digested brain material from hamsters i.c. inoculated with tissue from hamsters e.n. inoculated with mock infected (mock) or DY TME agents. The 19 and 21 kDa unglycoslyated PrP^Sc^ polypeptides are indicated on the left of the panel. o.b.–olfactory bulb; t.g.g.–trigeminal ganglion; c.l.n.–cervical lymph node; p.p.–peyer’s patch.
(TIFF)

S2 Fig Enriched PrP-res has similar PMCA seeding activity compared to prion-infected brain homogenate. Sypro Ruby analysis (A) and PMCA seeding activity of detergent enriched PK digested HY TME (B) and (C) DY PrP-res. Amount of PrP-res used for starting dilution was normalized to brain homogenate by Western blot. Arrow indicates migration of 27 kDa molecular weight marker. Bracketed regions indicate PrP-res banding patterns.
(TIFF)

S3 Fig Reduction in PrP^Sc^ abundance corresponds with reduction in PMCA seeding activity. PMCA conversion coefficient of PK digested brain homogenate. PMCA conversion coefficient was determined for proteinase K (PK) digested brain homogenate from (A) HY TME, (B) 139H or (C) DY TME-infected hamsters. The 19 or 21 kDa unglycoslyated PrP^Sc^ polypeptides are indicated on the left of the panel.
(TIFF)
