# Supplementary figures and images for: PrPSc formation and clearance as determinants of prion tropism
Source: PLoS Pathog. 2017 Mar 29;13(3):e1006298. doi: 10.1371/journal.ppat.1006298 (PMC5386299; doi:10.1371/journal.ppat.1006298)

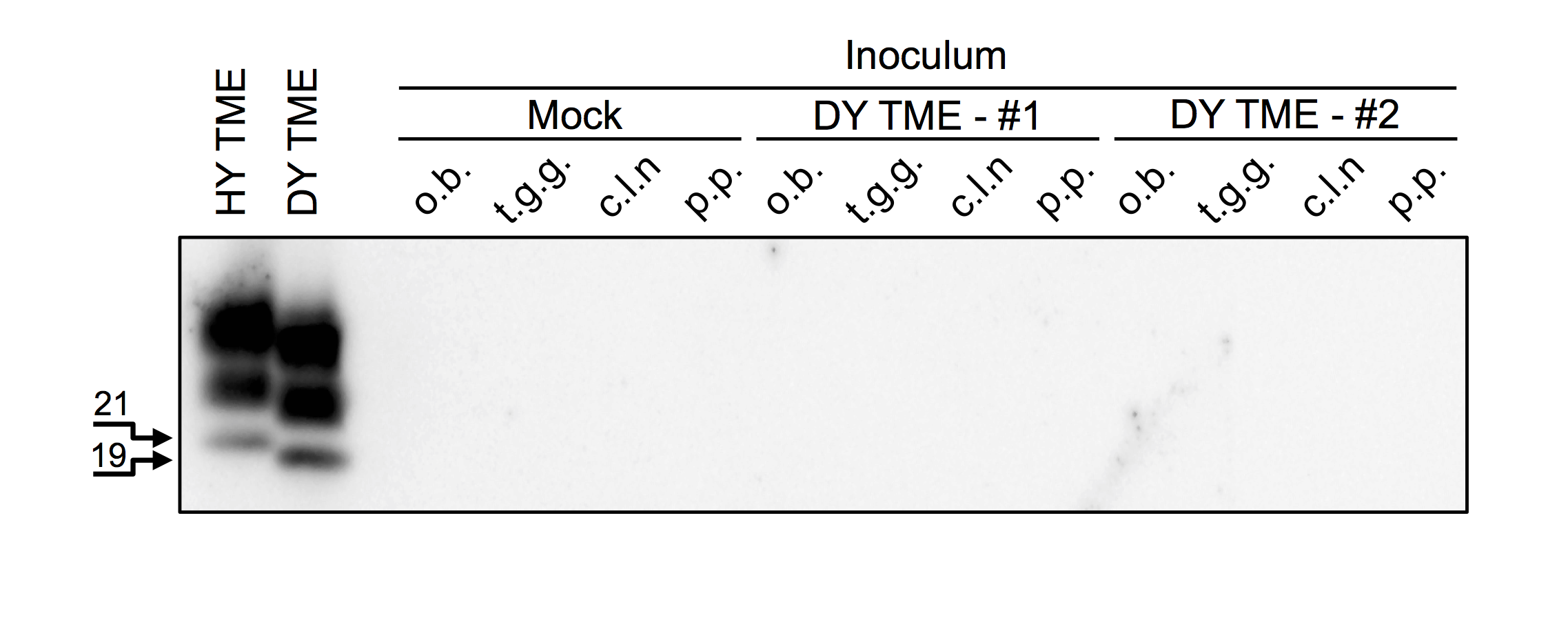

Supplement: S1 Fig — Western blot analysis of proteinase K digested brain material from hamsters i.c. inoculated with tissue from hamsters e.n. inoculated with mock infected (mock) or DY TME agents. The 19 and 21 kDa unglycoslyated PrPSc polypeptides are indicated on the left of the panel. o.b.–olfactory bulb; t.g.g.–trigeminal ganglion; c.l.n.–cervical lymph node; p.p.–peyer’s patch. (TIFF) [file ppat.1006298.s001.tiff]

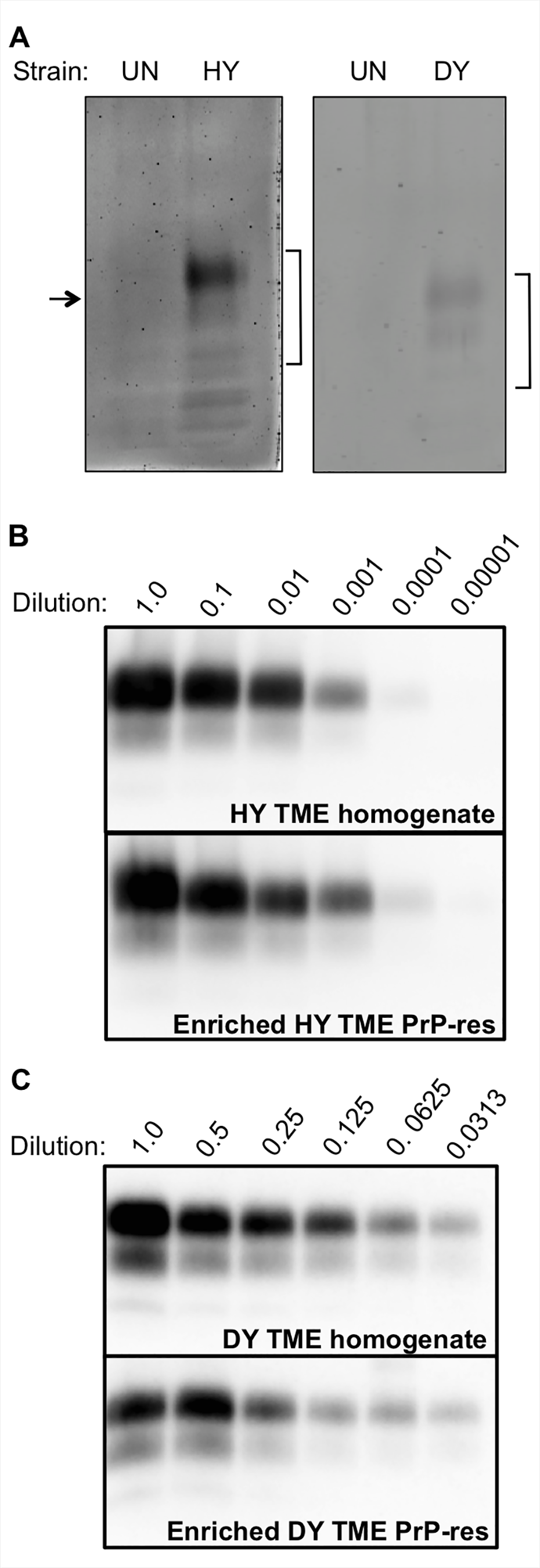

Supplement: S2 Fig — Sypro Ruby analysis (A) and PMCA seeding activity of detergent enriched PK digested HY TME (B) and (C) DY PrP-res. Amount of PrP-res used for starting dilution was normalized to brain homogenate by Western blot. Arrow indicates migration of 27 kDa molecular weight marker. Bracketed regions indicate PrP-res banding patterns. (TIFF) [file ppat.1006298.s002.tiff]

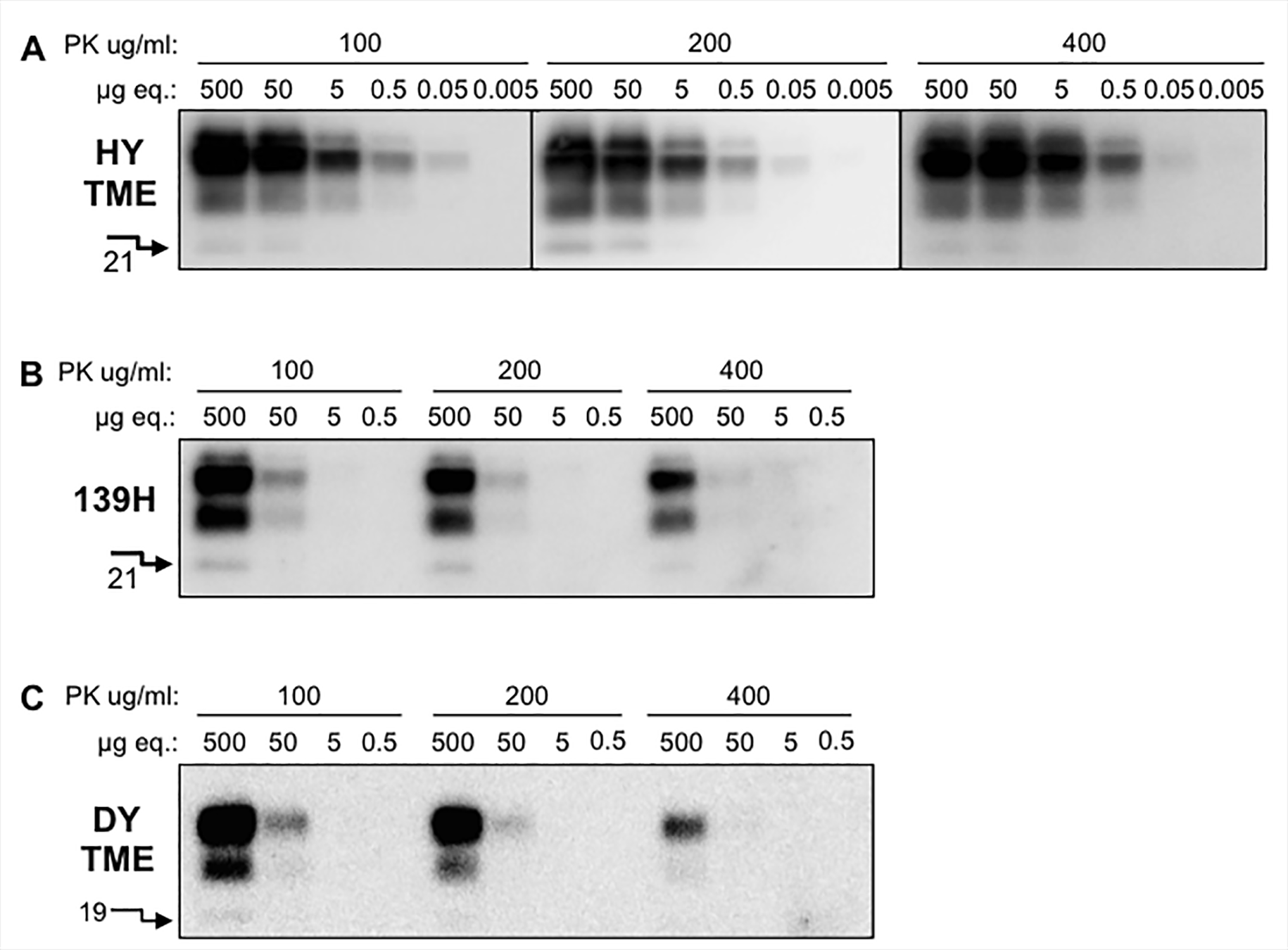

Supplement: S3 Fig — PMCA conversion coefficient of PK digested brain homogenate. PMCA conversion coefficient was determined for proteinase K (PK) digested brain homogenate from (A) HY TME, (B) 139H or (C) DY TME-infected hamsters. The 19 or 21 kDa unglycoslyated PrPSc polypeptides are indicated on the left of the panel. (TIFF) [file ppat.1006298.s003.tiff]
